# Robot-Beacon Distributed Range-Only SLAM for Resource-Constrained Operation

**DOI:** 10.3390/s17040903

**Published:** 2017-04-20

**Authors:** Arturo Torres-González, Jose Ramiro Martínez-de Dios, Anibal Ollero

**Affiliations:** Robotics Vision and Control Group, University of Sevilla, Escuela Superior de Ingenieros, c/Camino de los Descubrimientos s/n, 41092 Seville, Spain; arturotorres@us.es (A.T.-G.); aollero@us.es (A.O.)

**Keywords:** robot-sensor network cooperation, SLAM, sensor networks

## Abstract

This work deals with robot-sensor network cooperation where sensor nodes (beacons) are used as landmarks for Range-Only (RO) Simultaneous Localization and Mapping (SLAM). Most existing RO-SLAM techniques consider beacons as passive devices disregarding the sensing, computational and communication capabilities with which they are actually endowed. SLAM is a resource-demanding task. Besides the technological constraints of the robot and beacons, many applications impose further resource consumption limitations. This paper presents a scalable distributed RO-SLAM scheme for resource-constrained operation. It is capable of exploiting robot-beacon cooperation in order to improve SLAM accuracy while meeting a given resource consumption bound expressed as the maximum number of measurements that are integrated in SLAM per iteration. The proposed scheme combines a Sparse Extended Information Filter (SEIF) SLAM method, in which each beacon gathers and integrates robot-beacon and inter-beacon measurements, and a distributed information-driven measurement allocation tool that dynamically selects the measurements that are integrated in SLAM, balancing uncertainty improvement and resource consumption. The scheme adopts a robot-beacon distributed approach in which each beacon participates in the selection, gathering and integration in SLAM of robot-beacon and inter-beacon measurements, resulting in significant estimation accuracies, resource-consumption efficiency and scalability. It has been integrated in an octorotor Unmanned Aerial System (UAS) and evaluated in 3D SLAM outdoor experiments. The experimental results obtained show its performance and robustness and evidence its advantages over existing methods.

## 1. Introduction

This paper deals with Range-Only (RO) Simultaneous Localization and Mapping (SLAM) in which the robot uses range measurements to build a map of an unknown environment and to self-localize in that map. RO-SLAM does not require line-of-sight operation and is independent of lighting conditions, whereas visual SLAM is sensitive to them and is not suitable in the presence of dense dust or smoke [1]. Using radio beacons as landmarks naturally solves the data association problem, typical of visual SLAM. Besides, RO-SLAM operates with shorter measurement and state vectors than visual SLAM, resulting in significantly lower computational burden.

Our envisioned application is aerial robot autonomous navigation for maintenance and repairing in industrial plants. We are interested in RO-SLAM schemes where the robot uses nodes from a sensor network as landmarks. Consider a GPS-denied scenario where a large number of sensor nodes (beacons) have been deployed at unknown static locations. For instance, they have been placed at random locations for real-time monitoring an accident, or they are used for monitoring an industrial facility, and their exact location was not registered during deployment. This is not a constraint: sensor nodes are already used for monitoring and maintenance in many industrial plants. Each node periodically gathers and filters measurements and transmits them to a monitoring station. We assume that each node can measure the distance to other nodes. This is not a limitation, in fact most Commercial Off-The-Shelf (COTS) nodes can measure the Radio Signal Strength of Incoming packets (RSSI) and estimate the range to the emitting node [2]. Mapping and robot localization is essential for UAS navigation in the above GPS-denied environments. Besides, knowing the location of beacons enables robot-sensor network cooperative missions of interest in these scenarios such as sensor node transportation and deployment [3,4], collection of data from sensors [5,6] or node remote powering [7]. The potential capabilities of robot-sensor network collaboration have recently originated significant interest in RO-SLAM methods that use sensor nodes as beacons [8,9,10,11]. However, most existing techniques consider beacons as passive landmarks disregarding the computational, communication and sensing capabilities with which they are actually endowed.

The performance of RO-SLAM methods improves as the number of measurements integrated in SLAM increases. For instance, integrating beacon-beacon (inter-beacon) measurements improves map and robot estimation accuracy [12]. However, using more measurements requires spending more resources such as the energy, bandwidth and computational power required to gather, transmit and integrate the measurements. SLAM is a resource demanding task. In the above scenarios the robot and beacons perform many other tasks apart from SLAM, all of them sharing the available resources. Besides, the available resources can be, and usually are, constrained by technological or application limitations such as the available energy and computational capacity of beacons or the bandwidth of the sensing channel. Resource consumption reduction has attracted high interest in SLAM, but very few resource-constrained RO-SLAM methods have been reported.

This paper presents a RO-SLAM scheme that is capable of exploiting robot-beacon cooperation in order to improve SLAM accuracy and efficiency while meeting a given resource consumption bound. In this work, the resource consumption bound is expressed in terms of the maximum number of measurements that can be integrated in SLAM per iteration. The sensing channel capacity used, the beacon energy consumed or the computational capacity employed, among others, are proportional to the number of measurements that are gathered and integrated in SLAM. The proposed scheme can meet static and dynamic bounds, e.g., determined by an online resource allocation tool, enabling high flexibility, which can be of interest in many cases. Our scheme employs a distributed Sparse Extended Information Filter (SEIF) SLAM method, in which each beacon gathers and integrates in the SLAM update stage robot-beacon and inter-beacon measurements. Like SEIF algorithm, our scheme can be executed in constant-time. Our scheme also comprises a distributed tool that uses a gain-cost analysis to dynamically select the most informative measurements to be integrated in SLAM.

The proposed scheme has a robot-beacon distributed approach were beacons actively participate in measurement selection, gathering and integration in SLAM. It has the following main properties: (1) adaptive resource-constrained operation, since it dynamically adapts to satisfy the given resource consumption bound; (2) accuracy, since it integrates inter-beacon measurements, significantly improving map and robot localization accuracies and speeding up beacon initialization; (3) efficiency, since it gathers and integrates the most informative measurements and; (4) scalability, since all the involved tasks are executed in a distributed manner. This paper presents the scheme, evaluates and compares its performance with existing methods in experiments performed with an octorotor UAS.

The proposed scheme employs the distributed SEIF SLAM that we sketched in [13]. In this paper, it is enhanced and combined with a distributed measurement selection tool that enables resource-constrained operation. The main improvements in this paper over [13] are:

- development of a distributed robot-beacon tool that selects the most informative measurements that are integrated in SLAM fulfilling the resource consumption bound;
- harmonious integration of the distributed SEIF SLAM and the measurement selection tool. The resulting scheme outperforms that in [13], as described in Section 6;
- extension to 3D SLAM, integration and experimentation of the scheme with an octorotor UAS;
- new experimental performance evaluation and comparison with existing methods;
- new subsection with experimental robustness evaluation;
- extension and more detailed related work. Furthermore, the paper has been restructured and all sections have been completed and rewritten for clarity.

The paper is organized as follows. Section 2 summarizes the related work. Section 3 presents the problem formulation. Section 4 and Section 5 briefly describe the operation of the robot and beacons. Section 6 presents its evaluation, comparison and robustness analysis in 3D SLAM experiments. The conclusions are in Section 7.

## 2. Related Work

As it is known, in Simultaneous Localization and Mapping (SLAM) a robot builds a map of an unknown environment while simultaneously keeping track of its location within that map. RO-SLAM methods solve the SLAM problem using only range measurements. Recursive Bayesian Filters (RBFs) provide a solid statistical basis for estimation integrating measurements in presence of noise. A good number of RO-SLAM methods have been developed in the last years. Most of them employ approaches based on the Extended Kalman Filter (EKF) SLAM or on Fast-SLAM. These filters have been combined with different initialization tools in order to deal with the partial observability of range measurements. Particle Filters (PFs) [14] involve significant computational burden but provide accurate results. Probability grids [12,15] have also been used but are computationally inefficient to implement in 3D. Trilateration [9] is the simplest approach but it can lead to high initialization errors, which can originate inconsistent estimations. Gaussian mixtures [16,17] provide a multi-hypothesis representation that allows integrating measurements from the beginning in undelayed SLAM methods. Dual to Kalman Filters, Information Filters represent the state by its information vector ξ and its information matrix Ω. The Sparse Extended Information Filter (SEIF) [18] for online SLAM maintains a sparse representation of Ω. SEIF is naturally efficient in the update stage and the sparseness of Ω enables using efficient algorithms for motion update and state recovery. SEIF can be executed in constant time regardless of the map size.

Most RO-SLAM techniques employ only robot-beacon range measurements. Work [12] was the first in using also measurements between beacons (inter-beacon). Integrating inter-beacon measurements improves map and robot estimation accuracy and speeds up beacon initialization. Despite its advantages few methods employ them. Integrating more measurements involves higher consumption of energy, bandwidth and processing time to gather, transmit and integrate them in SLAM. Work [19] integrates inter-beacon measurements adopting decentralized methods to deal with the increase in the consumption of resources.

Efficiency in the use of resources has received high interest in SLAM. Some methods use decision making tools based on information metrics to balance efficiency and accuracy. Work [20] developed an approach that selects highly informative loop-closure links to simplify the Pose SLAM information form and reduce its computational cost. They also achieve a compact information matrix reducing the number of measurements by operating in open loop for long periods. This open loop operation requires good odometry estimations, which are not always available in all scenarios and applications. Work [21] presented a method for estimating the quality of visual measurements in single-camera SLAM maximizing the mutual information between measurements and states in order to select the best camera measurements. Despite its potential advantages, very few SLAM techniques, and even less RO-SLAM, for resource-constrained operation have been reported.

In work [22] we proposed a RO-SLAM scheme that improves efficiency by choosing between two measurement gathering modes. Mode selection is performed using a simple centralized tool based on heuristics of robot and map estimation uncertainty: It does not consider resource consumption requirements, hence it cannot adapt to given resource consumption bounds. Besides, mode decision is fully centralized at the robot, lacking scalability. The scheme proposed in this paper is capable of satisfying a given resource consumption bound. It uses a distributed measurement selection tool that prioritizes measurements analyzing cost and expected uncertainty improvement. Besides, the scheme is distributed and beacons actively participate in measurement selection, gathering and integration in SLAM.

### 2.1. Range Only SEIF SLAM in a Nutshell

This section briefly summarizes the SEIF SLAM algorithm as an introduction to the proposed scheme. Most expressions have been omitted. For notation simplicity, time subindex *t* has also been omitted. Refer to [23] for further details.

RO-SLAM methods commonly adopt a state vector $\vec{x}$ comprised of the robot position ($x_{r}$) and the location of all the beacons in the map ($x_{1},x_{2},\ldots ,x_{N}$). SEIF is based on Extended Information Filter (EIF). Duals to Kalman Filters, Information Filters (IFs) employ the canonical representation of multivariate Gaussian distributions. The standard representation is based on the mean vector μ and the covariance matrix Σ, whereas the canonical representation is based on the information vector $\xi =\Sigma^{-1}\mu$ and the information matrix $\Omega =\Sigma^{-1}$.

(1) $$\Omega =\left(\begin{array}{cccc} \Omega_{x_{r},x_{r}} & \Omega_{x_{r},x_{1}} & \cdots & \Omega_{x_{r},x_{N}}\\ \Omega_{x_{1},x_{r}} & \Omega_{x_{1},x_{1}} & \cdots & \Omega_{x_{1},x_{N}}\\ \vdots & \vdots & \ddots & \vdots \\ \Omega_{x_{N},x_{r}} & \Omega_{x_{N},x_{1}} & \cdots & \Omega_{x_{N},x_{N}} \end{array}\right)$$

The information matrix Ω is symmetric and positive-semidefinite. Each off-diagonal entry of Ω, called the link [18], represents the relation between two elements in $\vec{x}$. At any time in the SEIF SLAM operation some of the off-diagonal elements of Ω are zero meaning lack of information between the involved elements; some of them have high values, called strong links, meaning high amount of information; and a number of them have values close to zero, called weak links. Weak links have lower impact on the estimation of $\vec{x}$ than strong links but both involve similar computational burden. SEIF operates as EIF but maintains a sparse representation of Ω by keeping the number of active beacons bounded by a threshold. At each step the discovered beacons are classified in active and passive. Active beacons are those with non-zero links to the robot. Every time the number of active beacons is above the bound, the sparsification step is performed deactivating the beacons with the weakest links.

EIF measurement update modifies only the entries of Ω corresponding to the elements involved in the measurement. Factorizing Ω allows efficient update stages regardless of the map size. SEIF inherits this efficiency. Furthermore, by bounding the number of active beacons, SEIF significantly reduces the computational burden in the prediction stage. For linearizing the prediction and observation models it is necessary to recover the state estimate μ from the predicted $\bar{\Omega }$ and $\bar{\xi }$. SEIF recovers only the useful part of $\vec{x}$, robot and active beacons. Of course, enforcing sparseness in Ω involves an approximation error in the estimations obtained by SEIF. Work [18] suggests using sparsification bounds in the range [4,5,7,8,9,10,11] to balance between accuracy degradation and burden reductions.

### 2.2. Integration of Range Measurements

Range measurements have the problem of partial observability. To cope this it SEIF should be combined with an auxiliary beacon initialization mechanism. Our scheme uses PFs for beacon initialization due to their high flexibility. Each beacon can be in the “initialization phase” or in the “state vector phase”. In the “initialization phase” each beacon executes its own PF. When the PF converges, the beacon initializes its state vector: The “state vector phase” starts.

The observation model used for range measurement $z_{r,i}$ taken by the robot to beacon $b_{i}$ is:

(2) $$h_{r,i}\left(\mu \right)=\sqrt{\delta_{x}^{2}+\delta_{y}^{2}+\delta_{z}^{2}},$$

where $\delta_{x}=\mu_{x}-\mu_{x}^{i}$, $\delta_{y}=\mu_{y}-\mu_{y}^{i}$ and $\delta_{z}=\mu_{z}-\mu_{z}^{i}$. $\left(\mu_{x},\mu_{y},\mu_{z}\right)$ and ($\mu_{x}^{i},\mu_{y}^{i},\mu_{z}^{i}$) are respectively the estimated positions of the robot and of beacon $b_{i}$. $h_{r,i}$ is nonlinear. Its Jacobian is:

(3) $$H_{r,i}=\frac{\partial h_{r,i}}{\partial \mu }=\left[\begin{array}{cccccccc} \frac{\delta_{x}}{h_{r,i}} & \frac{\delta_{y}}{h_{r,i}} & \frac{\delta_{z}}{h_{r,i}} & \cdots & \frac{-\delta_{x}}{h_{r,i}} & \frac{-\delta_{y}}{h_{r,i}} & \frac{-\delta_{z}}{h_{r,i}} & \cdots \end{array}\right]$$

All the entries of $H_{r,i}$ that are not shown in (3) are zero. Only the entries of $H_{r,i}$ corresponding to the robot and beacon $b_{i}$ are not zero. The proposed method also integrates inter-beacon measurements, such as $z_{i,j}$ gathered by beacon $b_{i}$ to $b_{j}$. The adopted model $h_{i,j}$ is an extension of that in (2) taking $\delta_{x}=\mu_{x}^{i}-\mu_{x}^{j}$, $\delta_{y}=\mu_{y}^{i}-\mu_{y}^{j}$ and $\delta_{z}=\mu_{z}^{i}-\mu_{z}^{j}$. The entries of its Jacobian $H_{i,j}$ are zero except for those corresponding to the beacons involved in the measurement:

(4) $$H_{i,j}=\left[\begin{array}{cccccccccc} 0 & \cdots & \frac{\delta_{x}}{h_{i,j}} & \frac{\delta_{y}}{h_{i,j}} & \frac{\delta_{z}}{h_{i,j}} & \cdots & \frac{-\delta_{x}}{h_{i,j}} & \frac{-\delta_{y}}{h_{i,j}} & \frac{-\delta_{z}}{h_{i,j}} & \cdots \end{array}\right]$$

## 3. Problem Formulation

Consider a GPS-denied scenario where a number of sensor nodes endowed with sensing, computational and communication capabilities have been deployed at static locations. We assume that the location of the nodes is not known. For instance, they have been deployed at unknown locations or their exact location has not been registered during deployment. Each node is assumed equipped with a range sensor and can measure the distance to the robot or to other nodes within its sensing region. Range measurements are assumed affected by statistically independent Gaussian noise. We are interested in RO-SLAM techniques that use sensor nodes as radio beacons (landmarks), to online estimate the locations of sensor nodes and of the robot. The SLAM method can exploit the capabilities of the beacons deployed but, at the same time, should make efficient use of the available resources and should comply with resource consumption constraints.

**Figure 1:**
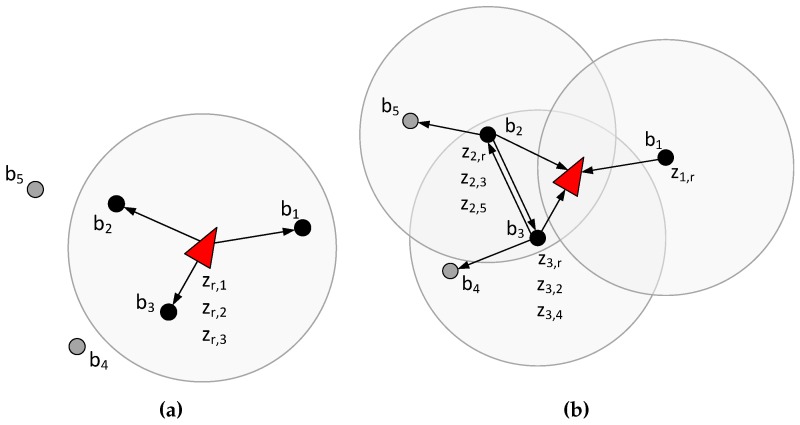
Measurement gathering in conventional SLAM methods (**a**) and in the proposed distributed SLAM scheme (**b**). Gray circles represent the sensing regions of the robot (**a**) and beacons $b_{1}$, $b_{2}$ and $b_{3}$ (**b**).

**Figure 2:**
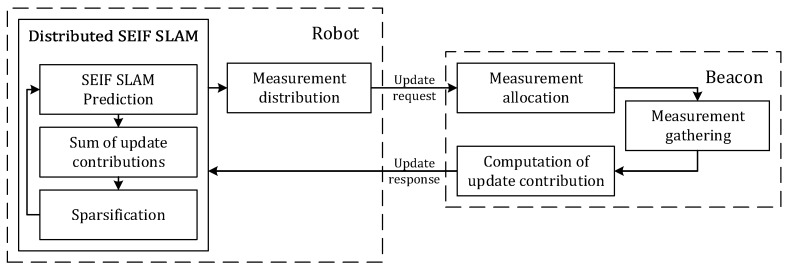
Diagram of the proposed resource-constrained RO-SLAM scheme.

In conventional RO-SLAM techniques the robot gathers range measurements to the beacons within its sensing region and integrates these measurements in the update stage, see Figure 1a. In the adopted scheme measurement gathering and integration in SLAM is distributed among the beacons. The robot computes the SEIF SLAM prediction stage and transmits the predicted state to the beacons within its sensing region. Each beacon receiving the message: (1) gathers range measurements to the robot and other beacons (inter-beacon measurements), see Figure 1b; (2) integrates these measurements and computes its contribution to the update stage and; (3) transmits its contribution to the robot. Finally, the update stage of SLAM expressed in the information form is additive [23] and the robot reconstructs the updated state by simply adding the contributions it received. The proposed scheme naturally exploits robot-beacon cooperation to solve online SLAM: Accurately, since it integrates inter-beacon measurements; efficiently, distributing measurement gathering and integration in SLAM, and hence sharing burden and energy consumption; and in a scalable manner.

Efficiency in the use of resources is very important in robot-sensor network cooperation. In most cases the SLAM algorithm is executed simultaneously with other tasks, all of them sharing the available resources. Furthermore, radio beacons gathering range measurements such as RSSI, Time of Arrival (ToA) or Differential ToA, make use of some kind of communication, which requires using a channel with a certain (constrained) capacity. In fact the capacity of the sensing channel is one of the most relevant constraints in settings with a high number and density of deployed beacons. In our problem resource consumption can be expressed in terms of the maximum number of measurements that are gathered and integrated in SLAM at each iteration. The use of the sensing channel, the consumption of beacons energy and of beacons computational capacity are proportional to the number of measurements that are gathered and integrated in SLAM. Hence, bounding the number of measurements constrains the consumption of the main resources involved.

**Figure 3:**
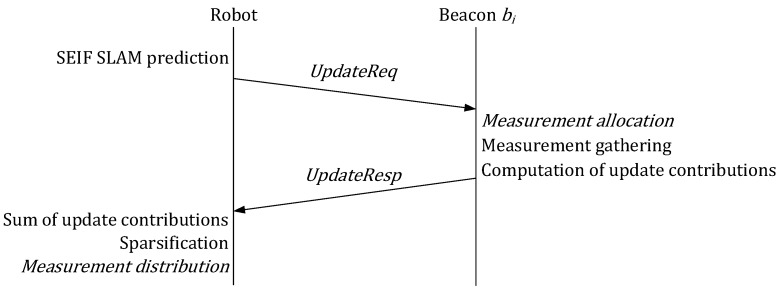
Operation and message interchange in the proposed scheme.

A diagram of the proposed scheme with its main modules is shown in Figure 2. The scheme combines (1) a distributed SEIF SLAM method in which beacons gather and integrate in SLAM robot-beacon and inter-beacon measurements; and (2) a distributed information-driven measurement selection tool that dynamically selects the measurements that are integrated in SLAM in order to improve performance while fulfilling the bound in the total number of measurements. Both components are executed in a distributed manner by the robot and by the beacons. Each beacon maintains a local version of the SLAM state whereas the global state is maintained only by the robot. The message interchange and the operation of the robot and beacons is summarized in Figure 3.

Methods that select the measurements that best reduce the uncertainty in the SLAM global state are necessarily centralized and have to deal with the information matrix of the global state. These methods incur in high computational burden with large maps and scale badly with the map size. The proposed scheme approximates this centralized measurement selection by a robot-beacon distributed tool that preserves the constant time execution and scalability. In Section 6.2 it is experimentally shown that the adopted tool is almost as accurate as the centralized measurement selection, adifference in map and robot RMS errors lower than 3%.

The distributed measurement selection tool is performed in two steps: *Measurement distribution* and *measurement allocation*. In *measurement distribution* the robot dynamically decides the number of measurements that are assigned to each beacon within the robot sensing region using expectations of uncertainty reductions. In *measurement allocation*, each beacon $b_{i}$ decides the actual measurements that it will gather and integrate in SLAM analyzing the cost and expected uncertainty improvement of integrating each measurement.

**Figure 4:**
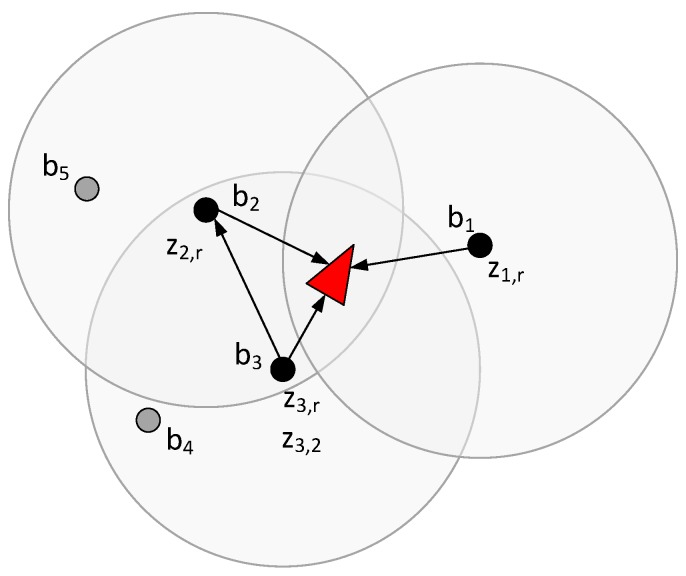
Measurement gathering with a low value of $NM_{max}$; $b_{1}$ gathers $z_{1,r}$; $b_{2}$ gathers $z_{2,r}$; $b_{3}$ gathers $z_{3,r}$ and $z_{3,2}$.

The bound in the number of measurements that can be gathered in each iteration is $NM_{max}$. As example where $NM_{max}$ has a high value is shown in Figure 1b: Each beacon within the robot sensing region gathers and integrates a measurement to each beacon within its sensing region. An example with a low $NM_{max}$ in shown in Figure 4. $b_{1}$ and $b_{2}$ are assigned with only one measurement and gather $z_{1,r}$ and $z_{2,r}$. $b_{3}$ is assigned with two measurements and besides $z_{3,r}$, it gathers $z_{3,2}$, the measurement that achieves the best expected uncertainty improvement-cost trade-off.

## 4. Operation of the Robot

The operation of the robot can be decomposed in four main tasks: (1) computation of the SEIF SLAM prediction stage; (2) reconstruction of the updated state using the contributions received by the robot; (3) computation of the sparsification step; and (4) *measurement distribution*. For brevity, most SEIF equations have been omitted. Refer to [23] for further details.

The robot operation is as follows, see Algorithm 1. First, the robot computes the SEIF SLAM prediction. Static beacons and nonlinear robot kinematic model are assumed. The robot Jacobian is computed at each time, which requires recovering the state. Our scheme uses the efficient algorithm described in [18] for motion update and state recovery. This algorithm computes the predicted information vector ${\bar{\xi }}_{t}$ and information matrix ${\bar{\Omega }}_{t}$ and recovers the predicted $\mu_{t}$.

**Algorithm 1:** Summary of the operation of the robot. **Require:**
$\xi_{t-1},\Omega_{t-1},NM_{max},LM$
1:SEIF motion update and state recovery2:Create and broadcast *UpdateReq* message3:Receive *UpdateResp* messages4:Extract $\xi_{i}$, $\Omega_{i}$ and $ui_{i}$ from *UpdateResp* messages5:Compute $\xi_{t}$ and $\Omega_{t}$ as in (6)–(7)6:SEIF Sparsification7:*Measurement distribution*. Create LM8:**return** $\xi_{t},\Omega_{t},LM$

As the robot moves beacons go in and out of the robot sensing region. The robot maintains $BS_{r}$, a list with the beacons that are currently within its sensing region. At each time *t* the robot broadcasts an *UpdateReq* message that includes $\mu_{t}$ and LM, a list created by the robot at *t* − 1 that contains the number of measurements that have been assigned to each beacon $b_{i}\in BS_{r}$. Transmitting the whole $\mu_{t}$ in the *UpdateReq* message is not suitable in cases with large maps. Only the elements in $\mu_{t}$ required for each beacon are transmitted. Let $ev_{i}$ be the vector with the estimates required by beacon $b_{i}$ to compute its contribution to the update stage:

(5) $$ev_{i}={\left[\begin{array}{ccc} \mu_{r} & \mu_{i} & \mu_{j} \end{array}\right]}^{T},$$

where $\mu_{r}$ is the estimation of the robot current location, $\mu_{i}$ is the estimation of the location of beacon $b_{i}$ and $\mu_{j}$ represents the estimations of the location of every beacon $b_{j}$ within the sensing region of $b_{i}$.

When $b_{i}$ receives the *UpdateReq* message, it performs as described in Section 5 and transmits to the robot an *UpdateResp* message with $\xi_{i,t}$ and $\Omega_{i,t}$, its contribution to the SLAM update stage. The robot reconstructs the updated state, $\xi_{t}$ and $\Omega_{t}$, using ${\bar{\xi }}_{t}$ and ${\bar{\Omega }}_{t}$ and the update contributions it received:

(6) $$\xi_{t}={\bar{\xi }}_{t}+\sum_{i}F_{i}^{T}\xi_{i,t},$$

(7) $$\Omega_{t}={\bar{\Omega }}_{t}+\sum_{i}F_{i}^{T}\Omega_{i,t}F_{i},$$

where $F_{i}$ is the projection matrix that implements the operations necessary to allocate $\xi_{i,t}$ and $\Omega_{i,t}$ at the suitable entries in $\xi_{t}$ and $\Omega_{t}$.

Next, the robot checks if $\Omega_{t}$ satisfies the SEIF sparsification bound. If not, the beacons with the weakest links are deactivated as described in [18].

The final step performed by the robot is to distribute the number of measurements $NM_{max}$ between $b_{i}\in BS_{r}$. $NM_{max}$ is considered an input to our scheme. It can be static or dynamic, computed by an online resource allocation tool, for instance analyzing the capacity of the channel using link quality estimators, see e.g., [24]. Measurement distribution is performed proportionally to $IG_{i,t}$, the usefulness of the measurements from beacon $b_{i}$ to reduce the uncertainty of the SLAM state. $b_{i}$ has impact on the SLAM state only if its update contribution reaches the robot, i.e., if $b_{i}$ receives the *UpdateReq* message sent by the robot and if the robot receives the *UpdateResp* message with the update contribution from $b_{i}$. These two events are statistically independent. Taking $p_{r,i}$ as the Packet Reception Rate (PRR) from the robot to $b_{i}$ and assuming symmetric PRRs, $IG_{i,t}$ can be estimated as:

(8) $$IG_{i,t}=p_{r,i}^{2}\;ui_{i,t},$$

where $ui_{i,t}$ estimates the capability of the measurements gathered by $b_{i}$ to reduce the uncertainty in the SLAM state. Transmission errors in sensor networks are not infrequent. This criterion naturally assigns more measurements not only to the most informative beacons, but also to those with better communication with the robot.

Each $b_{i}$ computes its own $ui_{i,t}$, described in Section 5, and transmits it to the robot in an *UpdateResp* message. The robot can measure $p_{r,i}$ to each $b_{i}\in BS_{r}$ by simply analyzing message transmission success. Next, the robot allocates the $NM_{max}$ measurements among beacons $b_{i}\in BS_{r}$ proportionally to $IG_{i,t}$ and creates LM, the list with the number of measurements assigned to each beacon $b_{i}\in BS_{r}$.

## 5. Operation of Beacons

The operation of beacons is summarized in Algorithm 2. Once beacon $b_{i}$ has received the *UpdateReq* message it performs as follows: (1) executes *measurement allocation* and selects the most informative measurements; (2) gathers and integrates in SLAM the selected measurements and; (3) transmits to the robot its update contribution in an *UpdateResp* message.

**Algorithm 2:** Summary of the operation of beacon $b_{i}$1:Receive *UpdateReq* message. Extract $ev_{i}$ and $LM_{i}$2:*Measurement allocation*. Compute $J_{i,j}$ and $ui_{i}$3:Gather the $LM_{i}$ measurements with the highest $J_{i,j}$. Create $MS_{i}$4:**if** ($b_{i}$ is at “state vector phase”) **then**5: Compute $\xi_{i,t}$ and $\Omega_{i,t}$ with $MS_{i}$ as in (15)–(16)6:**else**7: Use $MS_{i}$ to update the PF of $b_{i}$8:**end if**9:Create *UpdateResp* message and transmit it to the robot

### 5.1. Measurement Allocation

*UpdateReq* messages include LM. Once beacon $b_{i}$ has received an *UpdateReq* message, it extracts $LM_{i}$, the number of measurements it was assigned with. Let $BS_{i}$ be the set of beacons $b_{j}$ within the sensing region of $b_{i}$. In *measurement allocation* each beacon $b_{i}$ selects which measurements $z_{i,j},b_{j}\in BS_{i}$ it should gather and integrate in SLAM. We adopt a common approach in information-driven measurement selection and formulate the problem as the greedy optimization of a utility function that establishes a trade-off between information gain and resource consumption:

(9) $$J_{i,j}=r_{i,j}-\alpha c_{i,j}$$

$c_{i,j}$ is the cost of the resources consumed in gathering and integrating measurement $z_{i,j}$. In sensor networks energy is maybe the most constrained resource. We take $c_{i,j}$ as the energy consumed by $b_{i}$ in gathering and integrating $z_{i,j}$. $c_{i,j}$ could be different for each beacon, e.g., depending on the remaining energy in its batteries. For simplicity, $c_{i,j}$ was assumed the same for all measurements. The reward $r_{i,j}=ui_{i,j}$ is the expected SLAM uncertainty improvement resulting after integrating $z_{i,j}$. α is a weighting factor that balances the cost the reward. Its effects will be evaluated in Section 6.3. This cost-reward approach has been used in the literature, like in [25], where they used it to make decisions on a robot exploration problem. In our work, α was determined experimentally.

The reward $r_{i,j}=ui_{i,j}$ is determined as follows. In our distributed scheme each beacon maintains its own local state. ${\bar{\Omega }}_{i,t}$ is the predicted information matrix for time *t* of the local state of $b_{i}$. ${\bar{\Omega }}_{i,t}$ was computed by $b_{i}$ at *t* − 1. It is easy to notice that the updated information matrix of the local state of $b_{i}$ that would result after integrating measurement $z_{i,j}$ is:

(10) $$\Omega \prime_{i,t}={\bar{\Omega }}_{i,t}+\Omega_{i,j,t}$$

where $\Omega_{i,j,t}$ is the expected contribution of $z_{i,j}$ and is computed as follows:

(11) $$\Omega_{i,j,t}=H_{i,j,t}^{T}R^{-1}H_{i,j,t},$$

where *R* is the covariance matrix of the measurement noise and $H_{i,j,t}$ is the Jacobian of the observation model of measurement $z_{i,j}$ computed with $ev_{i}$, just received from the robot in the *UpdateReq* message.

On the other hand, in case of not integrating $z_{i,j}$, the updated information matrix for $b_{i}$ would be $\Omega_{i,t}^{n}={\bar{\Omega }}_{i,t}$. The uncertainty improvement $ui_{i,j}$ is the difference of the uncertainty in $\Omega \prime_{i,t}$ and in $\Omega_{i,t}^{n}$. Entropy is maybe the most widely-used metric for the uncertainty in a probability distribution. It is adopted in our scheme. Entropy can be used to measure the uncertainty of beacons in the “state vector phase” and also of beacons in the “initialization phase”, giving the same treatment to both cases. If beacon $b_{i}$ is in the “state vector phase”, its state follows a Gaussian probability distribution and its entropy can be computed using an exact expression. In this case $ui_{i,j}$ is as follows:

(12) $$ui_{i,j}=\frac{1}{2}\log\left(\frac{|{\bar{\Omega }}_{i,t}|}{|{\bar{\Omega }}_{i,t}+\Omega_{i,j,t}|}\right)$$

If $b_{i}$ is in the “initialization phase”, i.e., its PF has not converged, its probability distribution is approximated by the set of PF particles. In this case, there is not an exact expression and each $b_{i}$ computes its $ui_{i,j}$ using the approximate calculation described in [26].

It should be noticed that if $b_{j}$ is in the “initialization phase”, it is still not in the state vector of its neighbor $b_{i}$. Hence, $z_{i,j}$ is not useful to update the local map of $b_{i}$, either if $b_{i}$ is in the “initialization phase” or in the “state vector phase”. Hence, the uncertainty improvement $ui_{i,j}$ is taken as zero.

Long-term optimization of $J_{i,j}$ involves high computational burden and bad scalability. We adopted a simple but efficient greedy approach: At each time $b_{i}$ selects the $LM_{i}$ beacons $b_{j}\in BS_{i}$ that achieve the highest value in $J_{i,j}$. Of course, measurements with negative gain-cost utility, $J_{i,j}<0$, are not selected.

Each beacon $b_{i}$ receiving the *UpdateReq* message also computes $ui_{i}$, which will be used by the robot in *measurement distribution*. $ui_{i}$ estimates how good it is to assign measurements to $b_{i}$, i.e., the expected improvement in the uncertainty of the local state of $b_{i}$ if $b_{i}$ integrates one measurement to each beacon $b_{j}\in BS_{i}$. Similarly, the updated information matrix for $b_{i}$ that would result after integrating one measurement to each beacon $b_{j}\in BS_{i}$ is:

(13) $$\Omega {\prime \prime }_{i,t}={\bar{\Omega }}_{i,t}+\sum_{j\in BS_{i}}\Omega_{i,j,t}$$

As above, if no measurement is integrated, the updated information matrix is $\Omega_{i,t-1}$. Thus, $ui_{i}$ is computed as follows:

(14) $$ui_{i}=\frac{1}{2}\log\left(\frac{|{\bar{\Omega }}_{i,t}|}{|{\bar{\Omega }}_{i,t}+\sum_{j\in BS_{i}}\Omega_{i,j,t}|}\right)$$

### 5.2. Integration of Measurements

At this step, beacon $b_{i}$ has already gathered one measurement to the robot and to each of the beacons selected in *measurement allocation*. Let $MS_{i}$ be the set of gathered measurements. The next step is to integrate them. If beacon $b_{i}$ is in the “initialization phase”, it updates its PF with the measurements in $MS_{i}$. If $b_{i}$ is in the “state vector phase”, it integrates them in its local state and computes its update contribution as follows:

(15) $$\xi_{i,t}=\sum_{j\in MS_{i}}H_{i,j,t}^{T}R^{-1}\left[z_{i,j}-h_{i,j}\left(ev_{i}\right)+H_{i,j,t}ev_{i}\right],$$

(16) $$\Omega_{i,t}=\sum_{j\in MS_{i}}H_{i,j,t}^{T}R^{-1}H_{i,j,t},$$

where $h_{i,j}\left(ev_{i}\right)$ and $H_{i,j,t}$ are respectively the predictions and Jacobians for each measurement in $MS_{i}$, either robot-beacon or inter-beacon measurement. Finally, $b_{i}$ transmits an *UpdateResp* message to the robot with its contribution to the SEIF update ($\xi_{i,t}$ and $\Omega_{i,t}$) and to *measurement distribution* ($ui_{i}$).

## 6. Experiments

**Figure 5:**
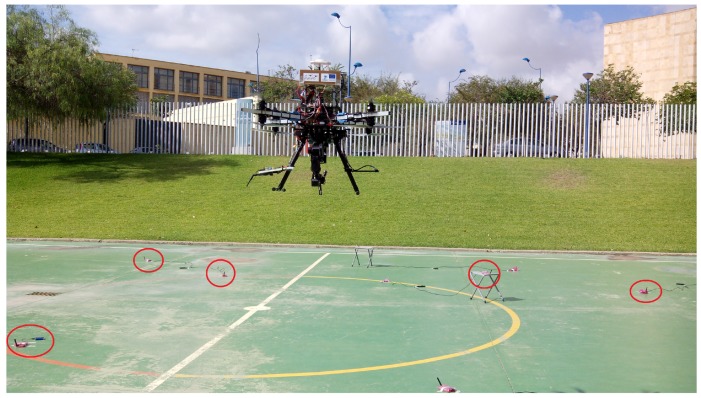
AMUSE UAS flying during one experiment at the School of Engineering of Seville.

The validation of the proposed scheme was performed in sets of 3D SLAM outdoor experiments with AMUSE UAS, an octorotor developed in the UE-FP7 ARCAS project for maintenance and repairing of industrial facilities [27], see Figure 5. Maintenance of industrial facilities is currently performed using sensor nodes that gather measurements for process monitoring and anomaly detection. In these complex scenarios GPS is often unavailable or has bad quality. UAS are suitable tools for confirming and eventually repairing the anomalies detected but they require accurate localization. The proposed resource-constrained RO-SLAM scheme is very interesting in this problem. Besides the typical technological constraints of UAS and beacons, in these scenarios there are often a high number of sensors and wireless devices involving significant bandwidth limitations. Besides, the energy consumed by nodes (beacons) is constrained in order to avoid frequent battery replacements.

**Figure 6:**
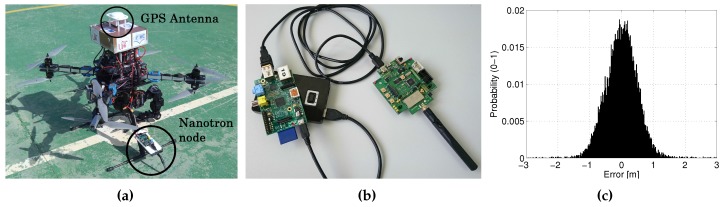
(**a**) Picture of AMUSE octorotor during one experiment; (**b**) Picture of a beacon comprised of a *Nanotron* ToF range sensor connected through USB to a *RaspberryPi* module powered by a battery; (**c**) Experimental outdoor characterization of *Nanotron nanoPAN 5375* sensors.

A total of 24 beacons were deployed at random locations and different heights in a 20 × 20 m scenario, beacons are marked in Figure 5. Each beacon was comprised of a *RaspberryPi* running Linux connected through USB to a *Nanotron nanoPAN 5375* [28] Time-of-Flight (ToF) range sensor and to a WiFi USB adapter, all powered by an external battery, see Figure 6b. The performance of *Nanotron* sensors in outdoors was characterized experimentally. Their range error can be modeled as a Gaussian PDF with zero mean and a standard deviation of $\sigma_{m}=0.6$ m, see Figure 6c. The resolution of these sensors is 0.01 m. It should be noticed that measurements from two or more different beacons can always be distinguished because the measurements are tagged with a unique identifier for each beacon. Each beacon run an independent ROS (Robots Operating System) node. The ROS node implements the beacon algorithm, gathers range measurements with the *Nanotron* ToF range sensor using an ad-hoc developed ROS driver and communicates with the other beacons using WiFi. One beacon was mounted on the landing skid of AMUSE, see Figure 6a. In the experiments the proposed SLAM scheme was executed at a rate of 1 Hz, one iteration per second. The robot beacon transmitted *UpdateReq* messages at a rate of 1 Hz. In these experiments the main resource constraint was imposed by the sensing channel bandwidth. $NM_{max}=80$ was the maximum number of range measurements per iteration that we could gather without interference in the experiments.

In these experiments AMUSE was in manual flight. The objective was not to use the proposed scheme for real-time navigation. AMUSE is equipped with a *Novatel OEM6* GPS unit with 2 cm accuracy. We used GPS only as ground truth for accuracy assessment. SLAM provides the generated map and robot location in a local coordinate frame. To compare with the ground-truth, an affine transform is performed on the final beacon locations, re-aligning the local solution into the same global coordinate frame. UAS odometry obtained from Inertial Measurement Units is often too noisy to be used in SLAM. Like most works in 3D SLAM, e.g., [29], we opted for employing some beacons, 5 in the experiments performed, as anchors for correcting the UAS localization.

The auxiliary PFs for beacon initialization were set with 500 particles. Each beacon executes its own PF, initializes the PF when it receives the first measurement and decides that it has converged when the maximum eigenvalue of the covariance matrix is lower than 0.4. The eigenvalues of the covariance matrix are directly proportional to the variance along the corresponding eigenvectors. Thus, the maximum eigenvalue is a measure of the volume (or at least the largest axis) of the confidence ellipsoid of the distribution. Using the eigenvalues puts the convergence condition on each axis. Our scheme has only two parameters: $NM_{max}$ and α. We used $NM_{max}=80$ and α=7.5 in all the experiments. For simplicity in these experiments the cost of measurement $z_{i,j}$ in $J_{i,j}$ was taken constant and equal to the energy consumed by beacons when taking one measurement $c_{i,j}=6.6$ mJ.

### 6.1. Validation

**Figure 7:**
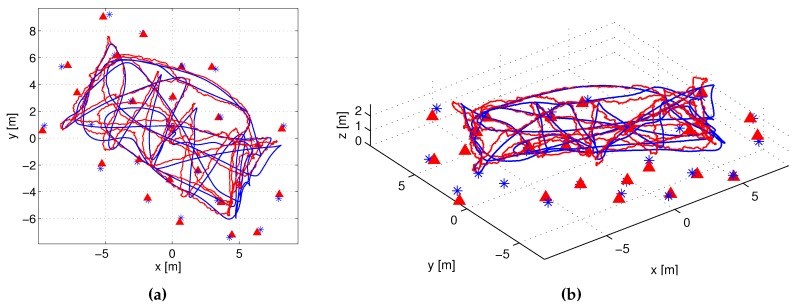
Results of the proposed scheme in a 3D SLAM experiment with AMUSE UAS: XY (**a**) and 3D views (**b**).

**Figure 8:**
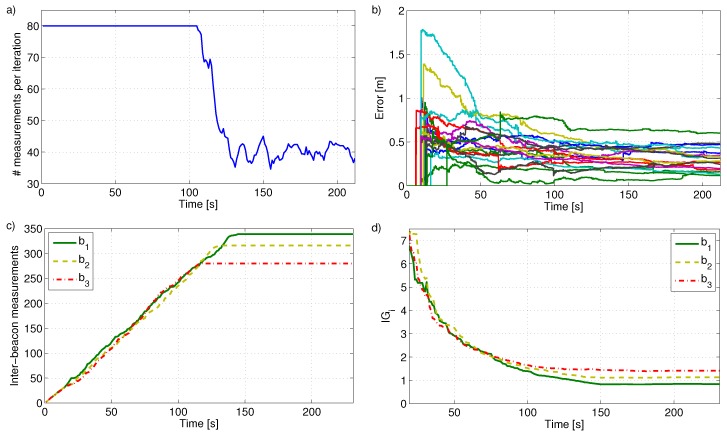
(**a**) Number of measurements integrated at each iteration along the experiment; (**b**) Evolution of beacon localization errors; (**c**) Number of measurements gathered by three beacons along the experiment; (**d**) Values of $IG_{i}$ along the experiment for the beacons in (**c**).

The result of the proposed scheme in one experiment in XY (left) and 3D (right) views is shown in Figure 7. Blue lines and stars represent respectively the ground truth UAS trajectory and beacon locations. Red lines and triangles are the resulting estimates. The total number of measurements integrated at each iteration along the experiment is shown in Figure 8a. At the beginning all beacons gathered all the measurements they were assigned with: In total $NM_{max}=80$ between all the beacons. As the experiment advanced the beacon local states had lower and lower uncertainty and inter-beacon measurements became less and less informative. From t=108 s on, some measurements achieved $J_{i,j}<0$; their reward was lower than the cost, and were not gathered anymore. In average the number of measurements per iteration in this experiment was 61, lower than $NM_{max}$. The proposed scheme ensures the given $NM_{max}$ bound avoiding reward-cost inefficient measurements.

**Figure 9:**
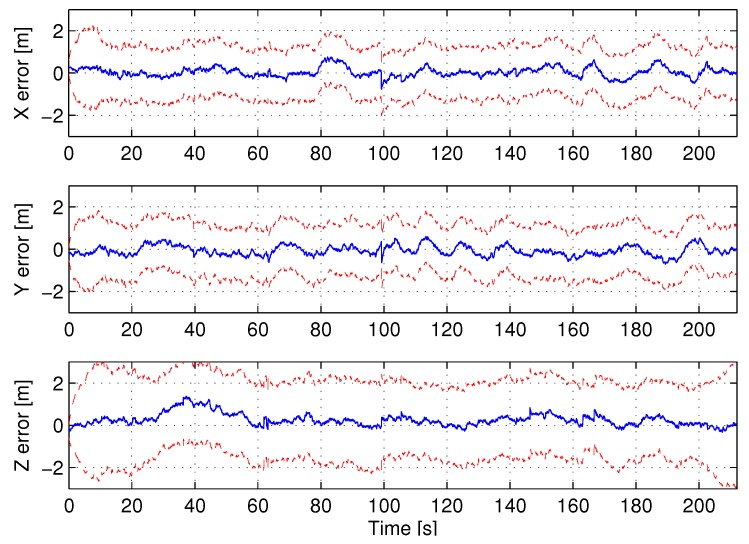
Evolution of UAS localization error in the experiment.

The evolution of beacon localization errors along the experiment is shown in Figure 8b. The drawing for each beacon starts when its PF converged. The majority of the PFs converged between t=6 s and t=16 s, shortly after the start of the experiment. The UAS localization errors in the three coordinates are shown in Figure 9. The red dashed lines represent the 3σ bounds showing the consistency of the estimations.

The cumulative number of inter-beacon measurements gathered by three beacons along the experiment is shown in Figure 8c. Similar curves were obtained for all beacons. The shape of each curve is a ramp with almost constant slope until the beacon stops gathering measurements. This evolution is useful to analyze the performance of *measurement distribution* and *measurement allocation*. At the beginning each beacon gathers all the measurements it is assigned with. *Measurement distribution* assigns measurements to $b_{i}$ proportionally to $IG_{i}$. More measurements are assigned to beacons with higher uncertainty. Hence, *measurement distribution* naturally balances the values of $IG_{i}$ of all the beacons. Figure 8d shows that the three beacons represented in Figure 8c have similar evolution in $IG_{i}$. This can be observed for all beacons. As a result all beacons are assigned with a similar number of measurements, resulting in similar slopes in Figure 8c. Beacon $b_{i}$ gathers and integrates $z_{i,j}$ as long as $J_{i,j}>0$. The uncertainty of $b_{i}$ will be lower as it integrates more measurements. After a while, the measurements gathered by $b_{i}$ will not satisfy $J_{i,j}>0$ and it will stop taking measurements, the lope becomes zero in Figure 8c. Each beacon has its own different situation (number of neighbors, time of PF convergence, etc.) hence, they will reach zero-slope at different times.

**Figure 10:**
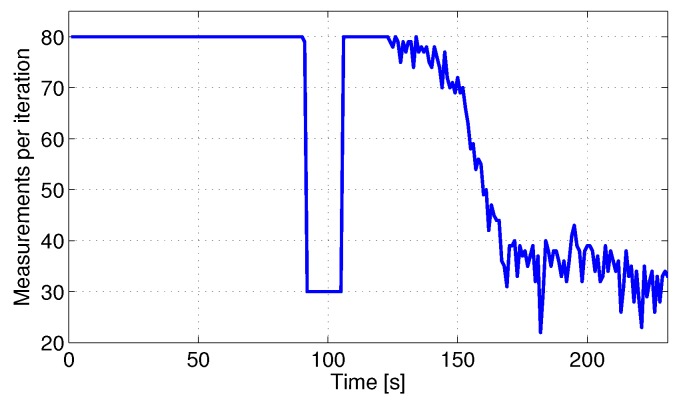
Experiment in Figure 8 taking $NM_{max}=30$ during t∈[90,105].

The proposed scheme can dynamically adapt to different values of $NM_{max}$. The previous experiment was repeated simulating that during interval t∈[90,105] the number of measurements was bounded by $NM_{max}=30$, see Figure 10. In this case the robot RMS error was 0.516 m, very similar to that with $NM_{max}=80$ along the entire experiment, which was 0.51 m. The difference in the map error was even smaller. The scheme selects the most informative measurements reducing the impact of changes in $NM_{max}$. The only effect is that beacons stop gathering inter-beacon measurements, reaching $J_{i,j}<0$, later in the experiment. They keep gathering measurements and at the end of the experiment the number of measurements integrated are the same in both cases.

### 6.2. Performance Comparison

The proposed scheme was evaluated and compared with other methods in 20 sets of real experiments with different beacon settings and UAS trajectories. Method *M1* is a conventional SEIF SLAM scheme that integrates only robot-beacon measurements. Method *M2* is the distributed SEIF SLAM reported in [13]. It integrates robot-beacon and all inter-beacon measurements. Method *M3* is *M2* combined with a tool that selects the $NM_{max}$ measurements that best improve the uncertainty in the global state: This tool is necessarily centralized at the robot. In the proposed distributed scheme each beacon selects the best measurements to improve its local uncertainty. Comparing with method *M3* allows evaluating how far our distributed measurement selection is from the centralized selection. The data from the sets of experiments was logged and the four methods were executed offline with the same parameters. Their performance is compared in Table 1, which analyzes robot and map RMS errors, convergence times of auxiliary PFs, number of measurements actually integrated per iteration, average energy consumed by beacons and average robot CPU time consumed evaluated in percentage w.r.t. that of *M1*. Recall that the number of measurements integrated per iteration is proportional to the energy consumed by beacons (shown in the table) and to the beacon computational time required for measurement integration (not shown in the table).

*M1* does not integrate inter-beacon measurements and hence had the poorest errors and PF convergence times. *M2* integrates inter-beacon measurements, which significantly reduces PF convergence times, 78%, and map and robot RMS errors, 32% and 16%, respectively, over *M1*. On the other hand, *M2* gathered and integrated 523% more measurements, which largely increased beacon energy consumption. *M2* distributes computation between the robot and beacons and hence reduces the robot CPU times over *M1*. *M3* is *M2* combined with a centralized tool that selects the $NM_{max}$ measurements that best improve the uncertainty of the global state. *M3* integrated 80 measurements per iteration, 61% lower than *M2*, and achieved similar RMS errors (difference <3%) and PF convergence times (<4%). However, it required much larger robot CPU times: It uses the information matrix of the global state to select the most informative measurements and computing determinants has $O(n^{3})$ complexity.

The proposed scheme obtained similar RMS errors, PF convergence times and robot CPU times to *M2* [13] requiring 70% less measurements and 70% lower beacon energy consumption. Besides, our scheme achieved similar RMS errors and PF convergence times to *M3* but required 23% less measurements (and beacon energy consumption). Each beacon uses the information matrix of its local state for measurement selection, requiring 78% lower robot CPU burden than *M3*. Besides, in our scheme each beacon maintains a local version of its map, which can be useful in some cases. Once beacons have built their local map, they can transmit it to any robot, which can immediately recover the full map applying map-joining techniques.

### 6.3. Discussion

In the following we discuss on the scalability of the proposed scheme and analyze the impact of transmission errors and of the parameters of the method: $NM_{max}$ and α.

The proposed method preserves the scalability of the distributed SEIF presented in [13]. *Measurement distribution* involves only the beacons within the robot sensing region, whereas *measurement allocation*, performed by each beacon $b_{i}$, involves only the beacons within the sensing region of $b_{i}$. Thus, the computational complexity depend on the beacon density, not on the map size. Beacons are used as landmarks in RO-SLAM: Highly inhomogeneous local beacon densities are not suitable in RO-SLAM. It is often more interesting if beacons are deployed in densities with some homogeneity, leading to constant time execution regardless of the map size.

$NM_{max}$ is taken as an input to our scheme. The performance of the proposed scheme with different values of $NM_{max}$ is summarized in Table 2. The measurements from all the experiments were logged and the proposed scheme was offline executed with $NM_{max}$. The integration of measurements is critical for PF convergence and low values of $NM_{max}$ decelerate PF convergence. On the other hand, the estimation accuracy was only slightly affected, which is attributed to its capability to select informative measurements. With $NM_{max}=80$ the average number of measurements actually integrated in SLAM was similar to that with $NM_{max}=60$. The explanation is the role of α. In the experiments all measurements are assumed to have the same cost. Thus, α acts as a threshold since measurement $z_{i,j}$ is gathered only if $J_{i,j}=r_{i,j}-\alpha c_{i,j}>0$.

The performance of the proposed scheme with different values of α is summarized in Table 3. α allows setting our scheme to prevent integrating measurements that are not very informative. With α=1.5, almost all measurements satisfy $J_{i,j}>0$ and the number of measurements integrated in SLAM is almost $NM_{max}$. With α=15, many measurements do not satisfy $J_{i,j}>0$ soon in the experiments and in average only 49.3 measurements were integrated per iteration. These were the two extremes in the range of α, an intermediate value α=7.5 was used. Despite the difference in the number of measurements, the value of α affects accuracy very slightly as shown in Table 3.

The proposed distributed scheme needs communication between the robot and beacons. In this sense the transmission errors in sensor networks cannot be ignored. Its performance assuming different PRR levels is summarized in Table 4. Our method explicitly considers PRR in the estimation of $IG_{i}$ and assigns more measurements to the beacons that have better link quality with the robot. As expected, it exhibits good robustness to PRR. Even with PRR = 40%, transmission error rate of 60%, its performance is very slightly perturbed.

## 7. Conclusions

RO-SLAM has some characteristics that make it more suitable than visual SLAM in a wide range of robot-sensor node cooperative missions. Most RO-SLAM methods consider beacons as passive landmarks and do not exploit the capabilities with which beacons are actually endowed. The accuracy of RO-SLAM estimations improves with the number and variety of measurements that are integrated in SLAM. However, using more measurements requires consuming more resources to gather, transmit and integrate the measurements in SLAM, which often contrasts with existing technological or application constraints.

This paper presents a scalable robot-beacon distributed RO-SLAM scheme for resource-constrained operation. The objective is to improve SLAM performance while meeting a given resource consumption bound expressed as the maximum number of measurements that can be integrated in SLAM per iteration. In our problem, the number of measurements is a good metric for resource consumption since it directly impacts the sensing channel capacity used, the beacon energy consumed and the computational capacity employed, among others.

The proposed scheme efficiently combines a distributed SEIF SLAM method that integrates robot-beacon and inter-beacon measurements, together with a distributed information-driven tool that selects the measurements to be integrated in SLAM balancing uncertainty improvement and resource consumption. The scheme has a robot-beacon distributed approach where beacons actively participate in measurement selection, gathering and integration in SLAM. Our scheme ensures resource-constrained operation with static or dynamic bounds, showing significant flexibility. It achieves higher accuracy and lower beacon initialization times than conventional SLAM methods. Besides, it can be executed in constant time regardless of the map size.

Its performance was validated and compared with existing methods in sets of 3D SLAM experiments. Robustness analysis confirmed its stable and predictable performance against transmission errors and different values of its parameters.

## Figures and Tables

**Table 1:** Comparison of the proposed scheme versus methods *M1*, *M2* and *M3*.

	*M1*	*M2*	*M3*	Proposed
Map RMS error (m)	0.49	0.33	0.34	0.34
Robot RMS error (m)	0.59	0.49	0.50	0.51
PF convergence times (s)	25.2	5.4	5.6	5.7
# of measurements/iteration	33.2	206.9	80	61.7
Beacon energy consumption (J)	43.7	272.2	105.2	81.1
Robot CPU time (% of *M1*)	100	65.6	265.5	58.6

**Table 2:** Average performance of the proposed scheme with different values of $NM_{max}$.

	${\mathrm{NM}}_{\max}=40$	${\mathrm{NM}}_{\max}=60$	${\mathrm{NM}}_{\max}=80$
Map RMS error (m)	0.35	0.346	0.34
Robot RMS error (m)	0.52	0.51	0.51
PF convergence times (s)	15.8	9.5	5.7
# of measurements/iteration	40	56.5	61.7

**Table 3:** Average performance of the proposed scheme with different values of α.

	α=1.5	α=7.5	α=15
Map RMS error (m)	0.34	0.34	0.37
Robot RMS error (m)	0.51	0.51	0.52
PF convergence times (s)	5.7	5.7	5.9
# of measurements/iteration	78.9	61.7	49.3

**Table 4:** Average performance of the proposed scheme with different PRR levels.

	PRR = 40	PRR = 60	PRR = 80	PRR = 100
Map RMS error (m)	0.4	0.37	0.35	0.35
Robot RMS error (m)	0.57	0.53	0.52	0.51
PF convergence times (s)	9.6	7.1	6.4	5.7
# of measurements/iteration	44.8	51.4	57.1	61.7

## Nomenclature

$\Sigma_{t}$	Covariance matrix of the SLAM global state at time *t*
$\mu_{t}$	Mean of the SLAM global state at time *t*
$\Omega_{t}$	Updated information matrix of the SLAM global state at time *t*
$\xi_{t}$	Updated Information vector of the SLAM global state at time *t*
${\bar{\Omega }}_{t},{\bar{\xi }}_{t}$	Predicted information matrix and predicted information vector of the SLAM global state for time *t*
$\xi_{i,t},\Omega_{i,t}$	Update contribution of beacon $b_{i}$ to $\xi_{t}$
$z_{r,i},z_{i,j}$	Measurement gathered by the robot to beacon $b_{i}$. Measurement gathered by beacon $b_{i}$ to $b_{j}$
$h_{r,i},h_{i,j}$	Observation models for robot-beacon and inter-beacon measurements
$H_{r,i},H_{i,j}$	Jacobians of the observation models for robot-beacon and inter-beacon measurements
$BS_{r},BS_{i}$	Sets of the beacons that are currently within the sensing region of the robot and beacon $b_{i}$, respectively
LM	List with the number of measurements assigned to each beacon in $BS_{r}$ in *measurement distribution*
$MS_{i}$	Set of measurements gathered by beacon $b_{i}$
$NM_{max}$	Maximum number of measurements that can be gathered and integrated per SLAM iteration
$J_{i,j}$	Utility function for measurement $z_{i,j}$
$r_{i,j},c_{i,j}$	Reward and cost for measurement $z_{i,j}$
α	Weighting factor between reward and cost
